# ECOLOGY AND WILDLIFE: Climate Change and the Arctic Diet

**DOI:** 10.1289/ehp.117-a292

**Published:** 2009-07

**Authors:** Erika Engelhaupt

**Affiliations:** **Erika Engelhaupt** is a freelance writer in Washington, DC, and has written for *Environmental Science & Technology, The Philadelphia Inquirer*, and other publications

Each spring, female polar bears and their cubs emerge from hibernation after months without food, and their survival depends on having good sea ice for hunting seals, their almost exclusive food. Also in spring, the Arctic sea ice begins to melt and break apart. Over the past 25 years, the timing of this melting has become less predictable as a consequence of warming in the Arctic, varying by more than a month. Researchers in Canada now report the first evidence that changes in the timing of the annual sea ice breakup have contributed to a dietary shift for polar bears from western Hudson Bay in the Canadian sub-Arctic. This shift may be accelerating the bears’ bioaccumulation of some classes of persistent contaminants, and people who consume these animals as part of a traditional subsistence diet could face greater exposure to contaminants that are passed up the food chain.

Research scientists Robert Letcher and Melissa McKinney of the National Wildlife Research Centre at Carleton University led the study, in collaboration with Elizabeth Peacock, a polar bear biologist for the Government of Nunavut. As reported in the 15 June 2009 issue of *Environmental Science & Technology*, the team measured levels of pollutants including polychlorinated biphenyls (PCBs) and polybrominated diphenyl ether (PBDE) flame retardants in western Hudson Bay polar bears between 1991 and 2007. They also measured fatty acids and carbon isotopes in the bears’ fatty tissues to determine what types of seals the bears were eating. Ice-associated seals, which eat from the sea floor, leave a different carbon signature than open-water seals, which eat higher on the food chain and thus accumulate higher contaminant levels.

In the years the sea ice broke up earlier, polar bears ate more open-water species such as harbor and harp seals instead of ice-associated species such as bearded seals, perhaps because the former are more abundant in light ice conditions. As evidenced by carbon isotope analyses, the timing of spring ice breakup explained 84% of the variation in the polar bears’ diet from year to year.

The researchers say the effects of the bears’ dietary shift are large enough in the case of PCBs to offset an apparent trend of decreasing concentrations in western Hudson Bay bears over the study period and to significantly accelerate an increasing trend in PBDEs. Letcher notes, however, that levels of PCBs, PBDEs, and other pollutants such as perfluorooctane sulfonate (PFOS) vary considerably among other northern bear populations and that time trend data are lacking in many regions. Climate change could also increase long-distance transport of pollutants to the Arctic by changing atmospheric circulation and hydrology, according to *Climate Change 2007: Impacts, Adaptation and Vulnerability,* the latest Intergovernmental Panel on Climate Change report.

The new research raises questions for Inuit populations that consume polar bears and other animals high on the food chain. “[The study] should be replicated because it suggests that changing polar bear diets could increase human exposure to persistent organic pollutants in Native Americans and others who consume polar bears as part of a traditional diet,” says epidemiologist Kristie Ebi, a Virginia-based independent consultant on health issues related to climate change. Ebi was a lead author of the human health chapter in *Climate Change 2007*, which concluded that the traditional diet of Arctic residents is likely to be adversely affected by climate change.

The Inuit diet, a varying mixture of fish and game, is rich in omega-3 fatty acids and has been linked to health benefits such as reduced heart disease, obesity, and diabetes. However, these foods are also the primary source of exposure to environmental contaminants for far-northern human populations. Rune Dietz, an environmental biologist at the University of Aarhus, Denmark, who studies contaminants both in polar bears and in human subsistence diets, says, “If we see [contaminant] increases in wildlife, most likely this will be related to hunters as well, because they have access to the same seals as polar bears.”

Shifting levels of other contaminants also are being observed. In ice-associated ringed seals, for example, changes in sea ice conditions increase mercury levels by shifting the seals’ food supply toward cod, which is the most contaminated of their food sources, according to a study led by Gary Stern and published in the 15 May 2009 issue of *Environmental Science & Technology*.

Contaminant levels are already of concern for the Inuit and are monitored by the Arctic Monitoring and Assessment Programme, which informs Arctic governments about pollution trends and sources. Russel Shearer, international chairman of the program, says, “The risk from exposure to contaminants from traditional foods needs to be balanced in the greater public health context, especially for women of childbearing age.”

## Figures and Tables

**Figure:**
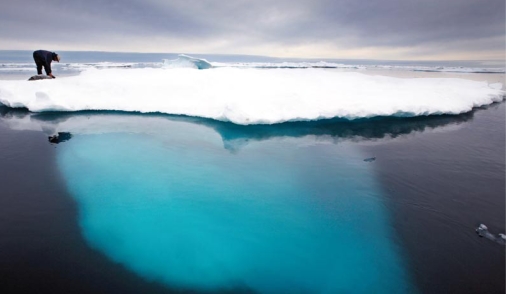
Inuit hunter and his seal prey, Ammassalik Island, Greenland, July 2007.

